# Inhibition of miR-33a-5p in Macrophage-like Cells In Vitro Promotes apoAI-Mediated Cholesterol Efflux

**DOI:** 10.3390/pathophysiology31010009

**Published:** 2024-02-28

**Authors:** Olanrewaju Oladosu, Emma Chin, Christian Barksdale, Rhonda R. Powell, Terri Bruce, Alexis Stamatikos

**Affiliations:** 1 Department of Food, Nutrition, and Packaging Sciences, Clemson University, Clemson, SC 29634, USA; oolados@g.clemson.edu (O.O.); echin@g.clemson.edu (E.C.); clbarks@g.clemson.edu (C.B.); 2 Clemson Light Imaging Facility, Clemson University, Clemson, SC 29634, USA; rhondar@clemson.edu (R.R.P.); terri@clemson.edu (T.B.)

**Keywords:** aortic smooth muscle cell, cardiovascular disease, immortalized cell line, reverse cholesterol transport, shRNA, trans-differentiation, vulnerable plaque

## Abstract

Atherosclerosis is caused by cholesterol accumulation within arteries. The intima is where atherosclerotic plaque accumulates and where lipid-laden foam cells reside. Intimal foam cells comprise of both monocyte-derived macrophages and macrophage-like cells (MLC) of vascular smooth muscle cell (VSMC) origin. Foam cells can remove cholesterol via apoAI-mediated cholesterol efflux and this process is regulated by the transporter ABCA1. The microRNA miR-33a-5p is thought to be atherogenic via silencing ABCA1 which promotes cholesterol retention and data has shown inhibiting miR-33a-5p in macrophages may be atheroprotective via enhancing apoAI-mediated cholesterol efflux. However, it is not entirely elucidated whether precisely inhibiting miR-33a-5p in MLC also increases ABCA1-dependent cholesterol efflux. Therefore, the purpose of this work is to test the hypothesis that inhibition of miR-33a-5p in cultured MLC enhances apoAI-mediated cholesterol efflux. In our study, we utilized the VSMC line MOVAS cells in our experiments, and cholesterol-loaded MOVAS cells to convert this cell line into MLC. Inhibition of miR-33a-5p was accomplished by transducing cells with a lentivirus that expresses an antagomiR directed at miR-33a-5p. Expression of miR-33a-5p was analyzed by qRT-PCR, ABCA1 protein expression was assessed via immunoblotting, and apoAI-mediated cholesterol efflux was measured using cholesterol efflux assays. In our results, we demonstrated that lentiviral vector-mediated knockdown of miR-33a-5p resulted in decreasing expression of this microRNA in cultured MLC. Moreover, reduction of miR-33a-5p in cultured MLC resulted in de-repression of ABCA1 expression, which caused ABCA1 protein upregulation in cultured MLC. Additionally, this increase in ABCA1 protein expression resulted in enhancing ABCA1-dependent cholesterol efflux through increasing apoAI-mediated cholesterol efflux in cultured MLC. From these findings, we conclude that inhibiting miR-33a-5p in MLC may protect against atherosclerosis by promoting ABCA1-dependent cholesterol efflux.

## 1. Introduction

Atherosclerosis is responsible for approximately 50% of all mortalities within developed nations [1]. Atherosclerosis causes narrowing of the arterial lumen which is the result of intimal cells accumulating excessive amounts of intracellular cholesterol [2]. Once intimal cells become lipid-laden, they convert into foam cells, which triggers both atherogenesis and exacerbates atherosclerosis [3,4]. There are two major types of intimal cells found in atherosclerosis lesions, which are monocyte-derived macrophages and MLC of VSMC origin.

The traditional perspective of atherosclerosis is that intimal lipid-laden foam cells predominantly originate from monocyte-derived macrophages [5]. However, more recent evidence indicates MLC of VSMC origin are also a source for intimal foam cells and foam cell populations may even contain a higher percentage of these types of MLC when compared to monocyte-derived macrophages [6,7,8]. The perplexity surrounding accurately identifying MLC versus monocyte-derived macrophages within intimal lesions is due to MLC of VSMC origin losing expression of their classical smooth muscle cell markers and instead exhibiting robust expression of classical macrophage markers [9]. However, lineage tracing studies have now shed light on appropriately identifying foam cell populations and have shown VSMC/MLC are a major cell type detected within atherosclerotic lesions [10].

A well-established trigger to induce VSMC trans-differentiation into MLC is cholesterol-loading VSMC [11]. We and others have also shown that removing cholesterol from MLC through apoAI-mediated cholesterol efflux restores VSMC phenotype within MLC of VSMC origin [12,13]. ApoAI is known to exclusively act as a cholesterol acceptor for ABCA1 [14] and so strategies to increase ABCA1 expression within MLC may result in atheroprotection via removing intracellular cholesterol through enhanced apoAI-mediated cholesterol efflux. The microRNA miR-33a-5p is well-recognized to silence ABCA1 expression [15]. Interestingly, miR-33a-5p is capable of silencing ABCG1 expression in rodents, but not in humans, while extensive published literature implies SR-BI to not be a likely target gene for miR-33a-5p [16,17,18,19]. Data has suggested that inhibiting miR-33a-5p may protect against atherosclerosis [17,18,20]. Much of the published work involving miR-33a-5p inhibition has involved cultured macrophages and we and others have shown that inhibiting miR-33a-5p in these cells results in increasing ABCA1-dependent cholesterol efflux [21,22,23,24]. A recent report from our laboratory has also shown that miR-33a deficient cultured MLC exhibit increased ABCA1-dependent cholesterol efflux [25]. While our results infer that inhibiting miR-33a-5p in MLC may be atheroprotective, the proof-of-principle approach we utilized also resulted in ablating expression of the other miR-33a mature strand, miR-33a-3p, and evidence implies this mature strand may be capable of silencing ABCA1 expression, too [26]. Therefore, to better determine whether miR-33a-5p expression in MLC attenuates ABCA1-dependent cholesterol efflux, expression of this specific mature strand of microRNA would need to be manipulated. In this study, we inhibited miR-33a-5p expression in cultured MLC to directly test whether inhibition of this mature strand would result in enhanced apoAI-mediated cholesterol efflux via ABCA1 upregulation.

## 2. Materials and Methods

### 2.1. Cell Culture and Maintenance

The immortalized VSMC line MOVAS cells are a commercially available cell line obtained from American Type Culture Collection (Manassas, VA, USA). We cultured MOVAS cells in standard growth medium [12] that consisted of the following ingredients: Dulbecco’s Modified Eagle’s Medium (DMEM; Corning, New York, NY, USA); penicillin–streptomycin (P/S; 1% final concentration; Corning), Fetal Bovine Serum; (FBS; 10% final concentration; Corning); geneticin (G418; 500 μg/mL final concentration; VWR Life Science, Radnor, PA, USA). MOVAS cells were maintained in 10 cm tissue culture dishes with incubation conditions set at 37 °C and 5% CO_2_. We washed MOVAS cells with PBS and replenished standard growth medium biweekly to allow cells to grow to a confluency of 70–90% before plating cells into treatment plates. Cells were allowed to reach a confluency of ~80% in respective treatment plates before initiating treatments.

### 2.2. Lentiviral Transduction

We used two lentiviral vectors to transduce MOVAS cells, with both vectors being purchased from VectorBuilder (Chicago, IL, USA). One lentiviral vector expresses a non-targeted scrambled control shRNA which served as the control lentivirus, while the other lentiviral vector expresses an antagomiR directed at miR-33a-5p. Both vectors also express GFP [27]. MOVAS cells were transduced with lentivirus (MOI = 5) for 24 h [27,28], washed with PBS, and utilized for subsequent experiments. To evaluate lentiviral transduction efficiency, transduced MOVAS cells were plated in four-chamber tissue-culture slides (Corning) and used for imaging analyses, which was conducted at the Clemson Light Imaging Facility (CLIF). Transduced cells were imaged by using a Leica DMi8 widefield microscope system (Leica Microsystems, Buffalo Grove, IL, USA), equipped with a Leica DFC9000 GTC camera and a 20× dry objective (N.A. = 0.4). To image signal from green fluorescent protein, we used a GFP-T filter cube with excitation wavelength of 475/40 nm and emission wavelength of 530/50 nm. All images were exported as .TIF using the same lookup table settings. The system software was Leica LAS-X, Version 3.6.0.20104 (Leica Microsystems).

### 2.3. Cholesterol-Loading MOVAS Cells to Induce MLC Trans-Differentiation

We converted MOVAS cells into a MLC phenotype by using the following protocol [12]. Briefly, we first washed cells with PBS, and then cultured cells in FBS-free DMEM which also contained 1% P/S, 2 mg/mL (final concentration) of fatty acid-free bovine serum albumin (BSA-FAFA; Sigma-Aldrich, St. Louis, MO, USA), and 10 μg/mL (final concentration) of cholesterol–methyl-β-cyclodextrin (MβCD:Chol; Sigma-Aldrich). To promote VSMC phenotypic switching to an MLC, we incubated MOVAS cells with this medium for 72 h. The rationale for using MβCD:Chol to induce lipid-loading instead of acLDL, oxLDL, or native LDL is because cultured VSMC are shown to be relatively resistant to cholesterol-loading and subsequent MLC trans-differentiation during prolonged incubation with LDL particles [11,13,29].

### 2.4. Western Blotting

Western blotting was performed as follows [25]. Briefly, transduced MOVAS MLC were washed with PBS and cell lysates collected with RIPA lysis buffer which contained protease inhibitors (VWR Life Science). Protein lysates were quantified with a BCA assay kit (VWR Life Science) and equal mass of protein samples were loaded on an SDS-PAGE gel and subjected to protein separation. We transferred separated proteins onto a PVDF membrane (Merck Millipore Ltd., Burlington, MA, USA), performed membrane blocking, and then probed for ABCA1 (1:1000 dilution, sc-58219; Santa Cruz Biotechnology, Dallas, TX, USA) and loading control (i.e., housekeeping protein) GAPDH (1:2000 dilution, sc-365062; Santa Cruz Biotechnology). After incubating PVDF membranes with primary antibodies, we incubated the membranes with an HRP-conjugated goat anti-mouse IgG secondary antibody (1:10,000 dilution, AP181P; Sigma-Aldrich). We incubated the membranes with ECL subtract (Immobilon ECL Ultra Western HRP Substrate; MilliporeSigma, Billerica, MA, USA) for detection of HRP and imaging was performed by using a ChemiDoc system (Analytik Jena US, Upland, CA, USA).

### 2.5. Cholesterol Efflux Assays

We used the following procedures to measure cellular cholesterol efflux [28]. Briefly, transduced MOVAS cells were trans-differentiated into MLC using our published protocol [12] also described above. During this 72-h incubation period, MOVAS cells were also exposed to [^3^H] cholesterol (1 μCi/mL; PerkinElmer, Waltham, MA, USA) which allows us to measure cholesterol efflux. After cholesterol-loading, we washed MOVAS MLC with PBS, and incubated cells with efflux medium which consisted of FBS-free DMEM, 1% P/S, and 2 mg/mL of BSA-FAFA. During this 72 h incubation step with efflux medium, MLC were also exposed to the cholesterol acceptor apoAI (100 μg/mL; Academy Bio-Medical Company, Houston, TX, USA) or vehicle only. After treatments, we filtered medium to remove non-adherent cells, washed MLC using PBS, and collected cellular extracts by using sodium hydroxide. We used a PerkinElmer Tri-Carb 4910TR liquid scintillation counter to count [^3^H] in both filtered medium and cellular extracts, and apoAI-mediated cholesterol efflux was then calculated as previously described [30].

### 2.6. RT-qPCR

Transduced MLC were initially washed with PBS and then lysed with TRI reagent (Zymo Research, Irvine, CA, USA). We subsequently extracted total RNA from lysed cells by using a Direct-Zol RNA purification kit (Zymo Research). After RNA harvest, we quantified the total isolated RNA with a SpectraMax^®^ QuickDrop™ Micro-Volume Spectrophotometer (MolecularDevices, LLC., San Jose, CA, USA). For RT-qPCR reactions involving small RNA quantification, we synthesized cDNA using a Takara Mir-X™ miRNA First-Strand cDNA Synthesis Kit (San Jose, CA, USA) and used this newly synthesize cDNA as template in our qPCR reactions which were amplified with a Takara TB Green^®^ Advantage^®^ qPCR Premix kit. We used a universal reverse primer provided with the Takara kits for our miR-33a-5p qPCR reactions along with the following forward primer (5′-CGCGTGCATTGTAGTTGCATTGC-3′). We used U6 as our housekeeping gene to normalize miR-33a-5p expression, with this primer pair being provided with the Takara kits (forward primer: 5′-GGAACGATACAGAGAAGATTAGC-3′; reverse primer: 5′-TGGAACGCTTCACGAATTTGCG-3′). To quantify mRNA in transduced MLC exposed to 100 μg/mL of apoAI diluted in efflux medium for 72 h, we followed the same RNA purification steps as described above, but synthesized cDNA from total RNA using a Quantabio qScript^®^ cDNA SuperMix kit (Beverly, MA, USA). The synthesized cDNA from mRNA was then used for amplification by using a Quantabio PerfeCTa SYBR Green Fastmix kit to quantify the following genes: SM-MHC (forward primer: 5′-AAGCTGCGGCTAGAGGTCA-3′; reverse primer: 5′-CCCTCCCTTTGATGGCTGAG-3′); F4/80 (forward primer: 5′-CTTTGGCTATGGGCTTCCAGTC-3′; reverse primer: 5′-GCAAGGAGGACAGAGTTTATCGTG-3′); GAPDH housekeeping gene (forward primer: 5′-CGTGCCGCCTGGAGAAAC-3′; reverse primer: 5′-TGGGAGTTGCTGTTGAAGTCG-3′). All qPCR reactions were quantified with a qTOWER3 G touch qPCR machine (Analytik Jena US) and gene expression was calculated by using the delta CT (ΔΔ^CT^) method [31].

### 2.7. Statistics

All statistical analysis was conducted with SigmaPlot (v15, Systat Software, Inc., San Jose, CA, USA). All our analyses assumed normality, based on Shapiro-Wilk testing. For *t*-testing, we conducted a Student’s *t*-test when equal variance was met, and a Welch’s *t*-test when equal variance was violated. Equal variance assumptions were based upon Brown-Forsythe testing. For all tests, we set significance of a *p*-value being <0.05.

## 3. Results

### 3.1. Transducing MOVAS Cells with LV-AntimiR33a5p Decreases miR-33a-5p Expression

**Figure 1:**
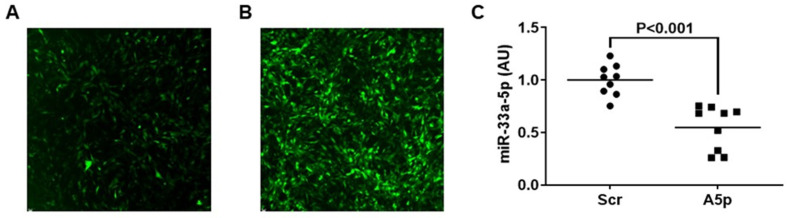
Lentiviral-mediated knockdown of miR-33a-5p in MOVAS MLC. (**A**,**B**) Transduction of MOVAS MLC with GFP-expressing lentivirus (LV) which also expresses either a non-targeting scrambled (Scr) control shRNA (**A**) or an anti-miR-33a-5p (A5p) antagomiR (**B**). (**C**) Expression of miR-33a-5p measured in MOVAS MLC transduced with either LV-Scr or LV-A5p. Data points are from three independent experiments that included three biological replicates from each independent experiment. Bars are group means. AU = arbitrary units.

We previously reported that lentivirus is capable of efficiently transducing MOVAS cells [28]. Therefore, we transduced control MOVAS cells with LV-Scr, and to inhibit miR-33a-5p, we also transduced cultured cells with LV-AntimiR33a5p. Since both lentiviral vectors also express GFP, we assessed transduction efficiency by immunofluorescence, and observed sufficient transduction in both groups of cultured cells (Figure 1A,B). Using qRT-PCR, we measured miR-33a-5p expression in transduced MOVAS cells subsequently converted into MLC via MβCD:Chol-loading and detected a significant decrease in miR-33a-5p expression with cells transduced with LV-AntimiR33a5p when compared to the control group of cultured cells (Figure 1C). These results indicate that LV-AntimiR33a5p demonstrates that capacity to inhibiting miR-33a-5p in VSMC/MLC.

### 3.2. Reduction of miR-33a-5p Expression in MOVAS MLC Results in Upregulation of ABCA1 Protein Expression

**Figure 2:**
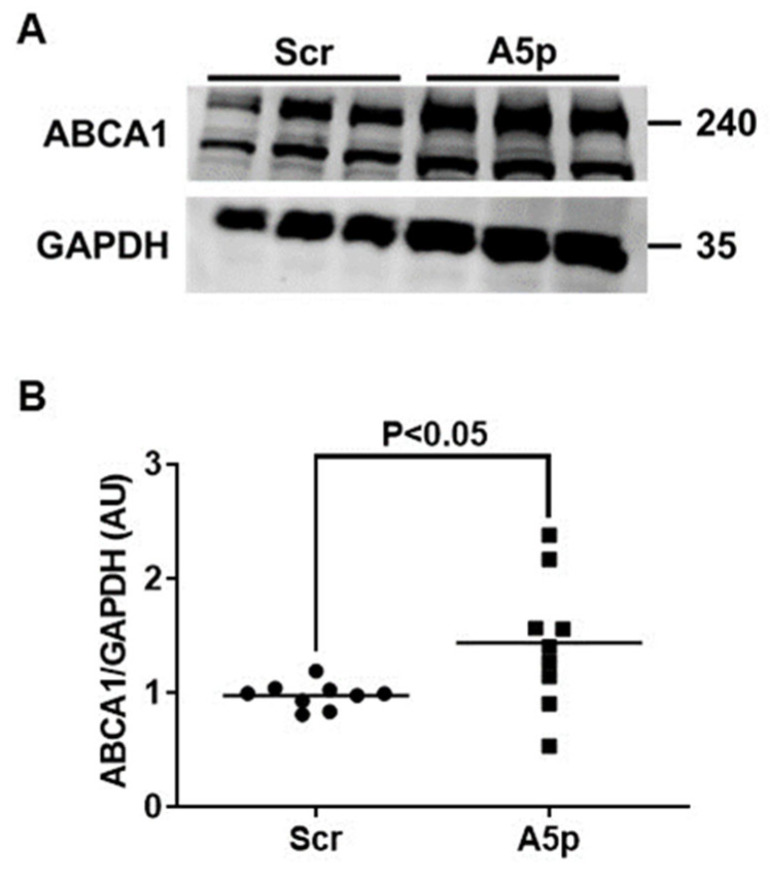
Decreasing miR-33a-5p expression in MOVAS MLC results in de-repression of ABCA1. (**A**) Representative western blot of ABCA1 and GAPDH for MOVAS MLC transduced with LV-Scr (Scr) or LV-AntimiR33a5p (A5p). (**B**) Quantitation of ABCA1 protein expression from (**A**) and two similar western blots. (**A**) Size markers are in kDa. (**B**) Data points are from three independent experiments which included three biological replicates for each independent experiment. Bars are group means. AU = arbitrary units.

We and others have shown that inhibiting miR-33a-5p in cultured monocytes/macrophages causes increased expression of ABCA1 [21,22,23,24]. However, to our knowledge, there is no published data on directly testing if precisely inhibiting miR-33a-5p in MLC of VSMC origin is capable of upregulating expression of ABCA1. Hence, we used immunoblotting to measure protein expression in MOVAS MLC that were transduced with either LV-AntimiR33a5p or LV-Scr. In these two groups of transduced cells, we observed a significant increase in ABCA1 protein expression from knocking down miR-33a-5p expression (Figure 2A,B). These findings imply that inhibition of miR-33a-5p in MLC may promote cholesterol removal through de-repression of ABCA1 protein expression.

### 3.3. Inhibiting miR-33a-5p in MOVAS MLC Enhances apoAI-Mediated Cholesterol Efflux Which Promotes Restoration of VSMC Phenotype

**Figure 3:**
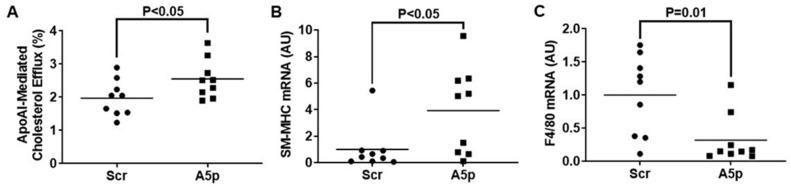
Reduction of miR-33a-5p expression in MLC of VSMC origin promotes VSMC restoration via enhancing apoAI-mediated cholesterol efflux. (**A**) Cholesterol efflux measured in MOVAS MLC transduced with either LV-Scr (Scr) or LV-AntimiR33a5p (A5p) upon exposure to apoAI. (**B**,**C**) Gene expression of the VSMC marker SM-MHC (**B**) and macrophage marker F4/80 (**C**) measured in MOVAS MLC transduced with Scr or A5p and subsequently exposed to apoAI. (**A**–**C**) Data points are from three independent experiments that include three biological replicates for each independent experiment. Bars are group means. (**B**,**C**) AU = arbitrary units.

ApoAI is a potent cholesterol acceptor that exclusively interacts with cell-surface ABCA1 to promote cellular cholesterol efflux [32]. Thus, increasing ABCA1 protein expression in MLC through miR-33a-5p knockdown may encourage the removal of intracellular cholesterol by enhancing apoAI-mediated cholesterol efflux. We measured apoAI-mediated cholesterol efflux in MOVAS MLC transduced with either LV-Scr of LV-AntimiR33a5p. In these two groups of transduced cells, we observed a significant increase in apoAI-mediated cholesterol efflux which occurred from miR-33a-5p inhibition when compared to the control groups of MOVAS MLC (Figure 3A). It has been proposed that restoring VSMC phenotype in MLC may be atheroprotective as VSMC-to-MLC trans-differentiation is thought to exacerbate atherosclerosis progression [33,34]. One well-established approach to revert cholesterol-loaded MLC to their original VSMC phenotype is by removing intracellular cholesterol. Therefore, we assessed whether the increase in ABCA1-dependent cholesterol that occurred from miR-33a-5p knockdown in cultured MOVAS MLC resulted in these cells to revert to VSMC in the presence of apoAI. When we measured mRNA expression of the classical VSMC marker smooth muscle myosin heavy chain (SM-MHC) [35] and classical macrophage marker F4/80 [36,37], we detected a significant increase in SM-MHC expression compounded with a significant decrease in F4/80 expression in MOVAS cells transduced with LV-AntimiR33a5p when compared to control cells (Figure 3B,C). Of note, under basal conditions SM-MHC is robustly expressed in VSMC while F4/80 expression should likely be weak (or absent), as F4/80 is notable as being expressed predominantly in mature macrophages [38,39]. Hence, our results suggest that inhibiting miR-33a-5p in MLC enhances apoAI-mediated cholesterol efflux and this effect is likely due to ABCA1 upregulation. Furthermore, this increase in apoAI-mediated cholesterol efflux may promote these cells to revert to VSMC via aiding to remove excess intracellular cholesterol.

## 4. Discussion

**Figure 4:**
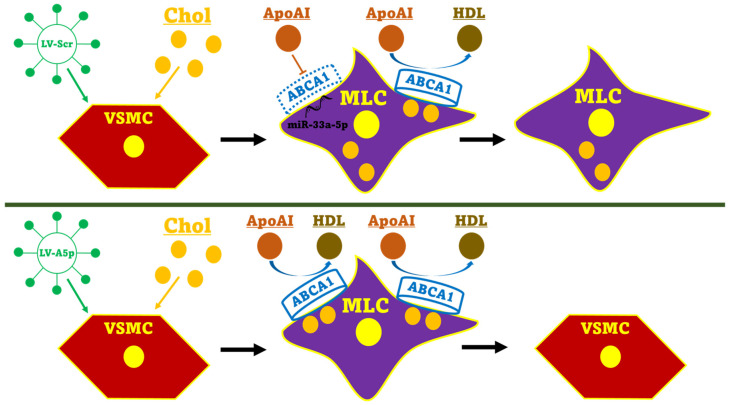
Schematic illustrating miR-33a-5p inhibition in MLCs of VSMC origin aids in reverting cells to VSMCs via enhancing apoAI-mediated cholesterol efflux. Top panel: Transducing VSMCs with LV-Scr causes cells to express a non-targeting scrambled shRNA. When these cells are loaded with cholesterol (Chol) to induce MLC trans-differentiation, miR-33a-5p reduces apoAI-mediated cholesterol efflux by silencing ABCA1 expression, resulting in cells to retaining an MLC phenotype. Bottom panel: Transducing VSMCs with LV-AntimiR33a5p (LV-A5p) causes cells to express anti-miR-33a-5p which inhibits miR-33a-5p expression. When cells are Chol-loaded to trigger MLC trans-differentiation, inhibition of miR-33a-5p promotes ABCA1 upregulation, resulting in increased apoAI-mediated cholesterol efflux that reverts cells to a VSMC phenotype.

In our study, we wanted to directly analyze whether precise inhibition of miR-33a-5p in MLC increases ABCA1-dependent cholesterol efflux. Using lentivirus, we transduced the VSMC line MOVAS cells with LV-AntimiR33a5p and then subsequently incubated the transduced MOVAS cells with MβCD:Chol to induce MLC trans-differentiation in these cells. Transducing these cultured cells with LV-AntimiR33a5p effectively inhibited miR-33a-5p expression, which resulted in upregulating protein levels of ABCA1. When we measured cholesterol efflux in these cultured cells using apoAI as the cholesterol acceptor, we detected enhanced apoAI-mediated cholesterol efflux, which was likely due to a significant increase in ABCA1 protein expression. We also observed inhibiting miR-33a-5p in cultured MLC of VSMC origin resulted in restoring VSMC phenotype in these cells as determined by increased SM-MHC mRNA expression compounded with suppressed F4/80 mRNA expression when these MβCD:Chol-loaded cells were also incubated with apoAI. Taken together, these findings demonstrate specific inhibition of miR-33a-5p in MLC of VSMC origin may be anti-atherogenic via removing intracellular cholesterol which has the capacity to restore VSMC phenotype and that these effects are from increased ABCA1-dependent cholesterol efflux (Figure 4).

MiR-33a-5p has arguably been the most characterized mature strand of microRNA within the context of atherosclerosis [19,40,41]. Since miR-33a-5p inhibition in cultured hepatocytes and macrophages results in increased ABCA1 protein expression and apoAI-mediated cholesterol efflux [17,18], it is speculative to assume this would lead to anti-atherogenic effects in vivo. However, administering anti-miR-33a-5p systemically has not consistently resulted in atheroprotection, even though this type of therapy does increase plasma HDL levels [20,42]. Moreover, chronic administration of anti-miR-33a-5p can lead to metabolic disturbances which include hypertriglyceridemia and hepatic steatosis [43]. With this in mind, directed therapy that precisely targets intimal MLC may be advantageous to safely deliver anti-miR-33a-5p to these cells, which may be a more effective treatment for atherosclerosis when compared to administering anti-miR-33a-5p systemically.

We chose lentivirus to introduce anti-miR-33a-5p to MOVAS cells, since we and others [28,44] have already shown lentiviral vectors efficiently transduce this cell line and cultured VSMC. As with our prior report [28], we observed effective gene manipulation when transducing MOVAS cells. However, quantification of lentiviral transduction efficiency using flow cytometry [45] would likely be needed when initial transductions in VSMC/MLC do not provide intended efficacy, to better gauge if higher lentiviral transduction efficiency may be achieved or if other viral vectors which demonstrate robust VSMC transduction efficiency [46] should be selected instead for enhancing transduction potential. Our strategy employing lentiviral transduction efficiency also conforms with previous research [23] that involved using lentivirus to effectively knockdown miR-33a-5p in cultured human macrophage cell lines. In this work [23], lentiviral vector-mediated miR-33a-5p inhibition resulted in upregulating ABCA1 expression and enhancing apoAI-mediated cholesterol efflux in cultured macrophages, which is similar to what we report. Of note, our findings utilize MLC which demonstrate behavior and phenotype that is vastly different from monocyte-derived macrophages [10,47]. However, the previous work from Min Mao and colleagues support miR-33a-5p expression possibly being linked to macrophage pro-inflammation [23], and so future work should be conducted to determine whether any pro-inflammatory effects of miR-33a-5p are also extended to include MLC, too.

Published data is currently scant on the possible atherogenic effects of miR-33a-5p expression in VSMC/MLC. We previously reported that inhibiting miR-33a-5p expression in primary VSMC does result in increasing ABCA1 protein expression and enhancing apoAI-mediated cholesterol efflux [48]. However, a recent report in our laboratory also showed that ablation of the miR-33a gene in the immortalized VSMC line MOVAS cells did not enhance apoAI-mediated cholesterol efflux, even though we did detect increased ABCA1 protein expression from miR-33a ablation [25]. When we MβCD:Chol-loaded miR-33a deficient MOVAS cells to induce MLC trans-differentiation though, we did observe increases in both ABCA1 protein expression and enhanced apoAI-mediated cholesterol efflux [25]. Since miR-33a deletion results in absent expression of both miR-33a-5p and miR-33a-3p, we cannot confidently infer from our previous published results whether the increase in ABCA1-dependent cholesterol efflux observed in MLC is actually due to miR-33a-5p deficiency, which led to us to performing this study. Since the results herein our report do show that inhibiting miR-33a-5p does promote ABCA1-dependent cholesterol efflux in cultured MLC, these findings demonstrate, proof-of-concept, that specific inhibition of miR-33a-5p in MLC may have therapeutic implications in treating atherosclerosis.

## 5. Conclusions

Our investigation is the first to our knowledge to confirm that inhibiting miR-33a-5p in cultured MLC increases both ABCA1 protein expression and apoAI-mediated cholesterol in these cells. Furthermore, we also show that inhibition of miR-33a-5p in MβCD:Chol-loaded MLC of VSMC origin promotes VSMC restoration in the presence of apoAI. Since we completed our experiments with immortalized cells, future studies should be devoted to loading cultured primary VSMC with MβCD:Chol to trigger MLC trans-differentiation, and then inhibiting miR-33a-5p within primary MLC. Utilizing this strategy to test similar end-points as described in our study may offer more physiologically applicable data, as data generated from immortalized cell lines sometimes do not correlate well in vivo [49]. In addition, regarding clinical implications, future experiments should knockdown miR-33a-5p within cultured primary human VSMC/MLC to attempt to recapitulate our findings when we inhibited miR-33a-5p in MOVAS cells, as data from human cells would likely offer a stronger perspective on whether miR-33a-5p inhibition precisely in human VSMC in vivo offers any potential therapeutic benefits. Moreover, while our current studies exclusively focused on inhibiting miR-33a-5p, some data does suggest that miR-33a-3p can also reduce ABCA1 expression [26], and so future experiments should consider inhibiting miR-33a-3p in MLC to directly assess whether inhibition of this microRNA may also result in increasing ABCA1-dependent cholesterol efflux. Lastly, for our findings to have any medical relevance, strategies that are able to precisely deliver anti-miR-33a-5p to intimal MLC would be needed. Our laboratory has recently developed a nanoparticle/exosome-based delivery system which may be capable of delivering anti-miR-33a-5p to sub-endothelial cells in vivo and thus far has shown promise during in vitro testing [24]. Experiments in our laboratory are currently underway which utilize this system to deliver anti-miR-33a-5p to cultured MLC to directly analyze whether anti-miR-33a-5p deliver to these cells can promote ABCA1-dependent cholesterol efflux in a similar manner to what we report in this study.

## Data Availability

All data represented in the study is contained within the manuscript.
